# Efficacy on knee function of Kinesio taping among individuals with anterior cruciate ligament reconstruction: A systematic review

**DOI:** 10.1371/journal.pone.0299008

**Published:** 2024-02-29

**Authors:** Peng Chen, Ling Wang, Wenxing Zhou, Lin Wang

**Affiliations:** 1 School of Exercise and Health, Shanghai University of Sport, Shanghai, China; 2 School of Sports Medicine, Wuhan Sports University, Wuhan, Hubei Province, China; 3 Sports Medicine and Rehabilitation Center, Shanghai University of Sport, Shanghai, China; 4 Shanghai Shangti Orthopaedic Hospital, Shanghai, China; Iran University of Medical Sciences, ISLAMIC REPUBLIC OF IRAN

## Abstract

**Objective:**

This study aims to evaluate systematically the efficacy of Kinesio taping (KT) on the knee function of individuals who undergo anterior cruciate ligament reconstruction (ACLR).

**Methods:**

This study was registered in PROSPERO (registration number CRD42023399885) on February 26, 2023. Randomized controlled trials (RCTs) about the effects on the knee function of KT among individuals after ACLR were electronically searched from PubMed, Web of Science, Embase, The Cochrane Library, and EBSCO from inception to July 02, 2023. The outcome measures included six continuous variables: quadriceps strength, hamstring strength, knee swelling, knee flexion angle, Lysholm knee function score, and Visual Analog Scale (VAS) pain scores. The Cochrane Risk Bias Assessment Tool was used to evaluate the quality of the included literature.

**Results:**

Seven RCTs including 278 patients who underwent ACLR were included in the systematic review. One of three (33%) studies found a remarkable increase in quadricep strength associated with the use of KT compared with the control group. Two of two (100%) studies found substantial increases in hamstring strength associated with KT. Two of four (50%) studies reported KT reduced knee swelling. Two of five (40%) studies reported considerable improvements in knee flexion angle in the groups that used KT. All three (100%) studies found KT did not improve Lysholm knee function scores. Three of four (75%) studies noted a significant reduction in VAS pain scores associated with KT.

**Conclusion:**

KT may help improve hamstring strength and reduce knee swelling and pain in patients after ACLR. Further studies are needed to determine the effects of KT on quadricep strength and knee flexion angle.

## 1 Introduction

As the primary static, dynamic stabilizing structure of the knee joint, the anterior cruciate ligament (ACL) plays a critical role in maintaining the stability and normal motion of the knee joint [1]. ACL rupture can lead to knee instability and decreased function, and is associated with secondary meniscal and cartilage injury [2]. Some evidence stated that anterior cruciate ligament reconstruction (ACLR) is the gold standard for regaining stability and improving knee function [3, 4]. Despite the tremendous advances in surgical techniques, nearly one-third of patients still do not return to their preinjury level of motion [5]. In addition, nearly one-quarter of young athletes experience a secondary ACL injury after returning to sport [6]. This outcome may be due to persistent post-traumatic neuromuscular inhibition, resulting in long-term deficits in muscle strength and motor function [7, 8]. Compared with primary ACLR surgery, the failure rate of ACL revision surgery is considerably higher, and the postoperative recovery of knee function is worse [9]. How to improve the muscle strength and motor function of patients after ACLR, and reduce the risk of secondary ACL injury has become a challenging problem in the field of sports medicine.

Different treatments were used to improve knee function in patients after ACLR, including neuromuscular functional training [10], neuromuscular electrical stimulation [11], whole-body vibration training [12], and blood flow restriction training [13]. Positive effects on biomechanical characteristics [10], postural control [12], muscle performance [11–13] of these treatments were confirmed. However, these treatment modalities have high equipment and technology requirements. In addition, due to the substantial financial burden, patients often cannot complete the whole cycle of treatment, which may adversely affect the treatment outcome [14]. Therefore, exploring safe, effective, economical, and convenient postoperative rehabilitation methods to minimize the family economic burden and improve the treatment effect is important.

Kinesio taping (KT) is the application of an adhesive elastic tape, which has been widely used in the field of sports medicine because of its advantages of economic convenience and noninvasiveness [15]. KT can be stretched by 130%–150%, and the elastic recoil in KT lifts the skin and increases the space between skin and muscle [16]. The tension provided by KT can increase blood circulation and lymphatic drainage, and reduce the pressure of swelling on subcutaneous pain receptors, thus relieving pain [17]. Some researchers assumed KT can facilitate and stimulate muscle function if its application starts at the origin of the muscle and ends at its insertion [18, 19]. In addition, the proprioceptive stimulation provided by KT promotes the recovery of neuromuscular control [20–22].

The above studies suggest KT may influence patients after ACLR [17, 20–22]. Some studies have investigated the effects of KT on muscle strength, joint stability, and pain and swelling in patients after ACLR [23–29]. However, the results in the existing studies on the clinical efficacy of KT on patients after ACLR are conflicting. Therefore, this study systematically evaluates the effect of KT on the rehabilitation outcomes of patients after ACLR to provide evidence-based medicine reference for the clinical use of KT in the treatment of postoperative ACLR.

## 2 Data and methods

### 2.1 Literature search methods

This study was enrolled in the International Prospective Register of Systematic Reviews on February 26, 2023 (registration number CRD42023399885) and completed in accordance with the PRISMA checklist. Randomized controlled trials (RCTs) about the effects on knee function of KT on individuals after ACLR were electronically searched from PubMed, Embase, The Cochrane Library, Web of Science, and EBSCO databases according to PRISMA (reporting items for systematic reviews and meta-analysis) guidelines using the following search terms: “Anterior Cruciate Ligament”, “Anterior cruciate ligament reconstruction”, “Anterior Cruciate Ligament Injuries”, “ACL”, “ACLR”, “ACL Injury”, “Athletic Tape”, “Kinesio taping”, “Kinesio Tape”, and “Kinesiotape”. Searches were conducted by two independent researchers following the search formula. No limit on the year of publication was set, and the final search was updated to July 02, 2023.

### 2.2 Inclusion criteria

The inclusion criteria of the current study included the following (1) Study type: RCT; (2) Study population: patients who underwent ACLR, without restriction on gender, age, or graft type; (3) Interventions: control group with blank control, sham KT, or conventional rehabilitation; KT group with KT alone or based on the control group; (4) Findings included at least one of the following: quadricep strength, hamstring strength, knee swelling, knee flexion angle, Lysholm knee function score, and Visual Analog Scale (VAS) pain score; (5) Language: English.

### 2.3 Exclusion criteria

The exclusion criteria of the current study included the following: (1) Studies lacking outcome data, (2) Conference papers, (3) Duplicate studies, (4) Studies with serious flaws in experimental design, and (5) Studies with low quality or incorrect data.

### 2.4 Risk of bias assessment

Quality assessment was evaluated using the Cochrane Risk Assessment Scale that consists of seven domains: (1) random sequence generation, (2) allocation concealment, (3) experimental staff blinding, (4) result staff blinding, (5) data completeness, (6) selective reporting of results, and (7) other sources of bias. For each source of bias, studies will be classified as having a low, high or unclear (if reporting was not sufficient to assess a particular domain) risk. Two independent reviewers evaluated the results. In case of disagreement, differences of opinion were discussed and resolved with a third author.

### 2.5 Data extraction

Two investigators (LW and WZ) screened the literature and extracted relevant data using an independent double-blind method based on the inclusion and exclusion criteria. Screening was first performed based on the title and abstract to exclude irrelevant literature. Then, the full text was further read to decide whether to include the literature. In case of disagreement, a third researcher participated in decision making. The following data were extracted from each included study: first author, year, sample size, age, gender, surgical graft, intervention time, follow-up time, intervention program, KT protocol, outcome indicators, and adverse effects. If the original text did not include the target data or the information was equivocal, an email was sent to contact the corresponding author for help or confirmation.

### 2.6 Statistical analysis

The findings were qualitatively synthesized using the following steps: (1) Data in the selected studies were assessed, contrasted, compared, and summarized in a table (Tables 1 and 2). These data included the sample, age, sex, intervention time, graft type, intervention program, KT protocol, and main findings. (2) Similarities and differences between the main findings of the selected studies were highlighted. The risk of bias due to missing results was minimized by inclusion of results from sources.

**Table 1:** Basic characteristics of the included literature.

Author, Year	Country	Sample size	Age	Intervention time	Graft type
Balki 2016 [23]	Turkey	KT: 15 (15M)	KT: 28.60±4.50	Day 5 after surgery	KT: Ham 9, Allograft 6
		Con: 15 (15M)	Con: 27.66±7.45		Con: Ham autograft 12, Allograft 3
Baltaci 2021 [24]	Turkey	KT: 28 (28M)	KT: 40.15±14.40	Day 2 after surgery	KT: Ham autograft 28
		Con: 28 (28M)	Con: 40.11±10.43		Con: Ham autograft 28
Boguszewski 2013 [25]	Poland	KT: 13	20–41	Not reported	Not reported
		Con: 13			
Chan 2017 [26]	Singapore	KT: 30 (22M, 6F)	KT: 27.4±8.25	Within one week after surgery	Not reported
		Con: 30 (24M, 6F)	Con: 26.3±7.04		
Khabazan 2017 [27]	Ireland	KT: 12	Not reported	Within six months after surgery	KT: Allograft 12
		Con: 12			Con: Allograft 12
Labianca 2022 [28]	Ireland	KT: 26 (26M)	KT: 28.5±5.30	Day 2 after surgery	KT: Ham autograft 26
		Con: 26 (26M)	Con: 29.2±4.60		Con: Ham autograft 26
Oliveira 2016 [29]	Brazil	KT: 15 (15M)	28.6±3.8	Between 12–17 weeks after surgery	KT: Ham autograft 15
		Con: 15 (15M)			Con: Ham autograft 15

KT: Kinesio taping, Con: Control, Ham: Hamstring, M: male, F: female

**Table 2:** Basic characteristics of interventions included in the literature.

Author, Year	Intervention program	KT protocol	Outcome indicators	Adverse reactions
Balki 2016 [23]	KT: KT plus routine rehabilitation, KT was applied twice for 10 days. Tapes were changed every five days. Con: Routine rehabilitation and sham tape.	The Y-shaped tape was used for quadriceps and hamstrings with 25%–50% tension. The base of the fan-tapes was placed toward the closest lymph nodes with 15% tension.	quadriceps strength→	None
			hamstring strength↑	
			knee flexion angle↑	
			Lysholm knee function score→	
Baltaci 2021 [24]	KT: KT plus routine rehabilitation, KT was applied three times for three days. Tapes were changed every day. Con: Routine rehabilitation.	First, the tape was adhered without tension, covering the middle of the patella with a grid-like appearance. The second KT was applied to the medial side of the knee, and the third KT was applied to the lateral side, intertwining one another.	VAS pain score→	Not reported
			knee swelling→	
			knee flexion angle→	
Boguszewski 2013 [25]	KT: KT plus routine rehabilitation, KT was applied four times for four weeks. Tapes were changed every seven days. Con: Routine rehabilitation.	The Y-shaped tapes was used for quadriceps with 15%–50% tension. I-shaped tapes were placed laterally and medially on the lower limb. The medial part of the tape, starting from the tibial tuberosity up to the femoral condyles, was stretched between 75%–100%.	VAS pain score↓	Not reported
			knee swelling↓	
			knee flexion angle↑	
Chan 2017 [26]	KT: KT plus routine rehabilitation, KT was applied twice for two weeks. Tapes were changed every five days. C: Routine rehabilitation.	The tape was laid over the knee in a basket-weave manner at 10% tension.	VAS pain score↓	Two subjects developed a mild allergy.
			knee swelling→	
			knee flexion angle→	
			Lysholm knee function score→	
Khabazan 2017 [27]	KT: KT was applied for 24 hours. Con: No taping.	The tape was used from origin to insertion of the quadriceps with 50% tension, around and below the patella bone.	quadriceps strength↑	Not reported
			hamstring strength↑	
Labianca 2022 [28]	KT: KT plus routine rehabilitation, KT was applied four times for four weeks. Tapes were changed every five days. Con: Routine rehabilitation.	The Y-shaped tape was used for quadriceps with 40% tension. Two more strips were cut into five bands and applied to the popliteal fossa without tension.	VAS pain score↓	None
			knee swelling↓	
			knee flexion angle→	
			Lysholm knee function score→	
Oliveira 2016 [29]	KT: KT was applied during testing. Con: No taping.	Three I-shaped tape was used for quadriceps with 50% tension.	quadriceps strength→	None

KT: Kinesio taping, Con: Control, VAS: Visual Analog Scale, ↑: The result of the KT group was substantially higher than that of the control group; →: No substantial difference was noted between the KT group and the control group; ↓: The result of the KT group was considerably lower than that of the control group.

## 3 Results

### 3.1 Study selection

**Fig 1:**
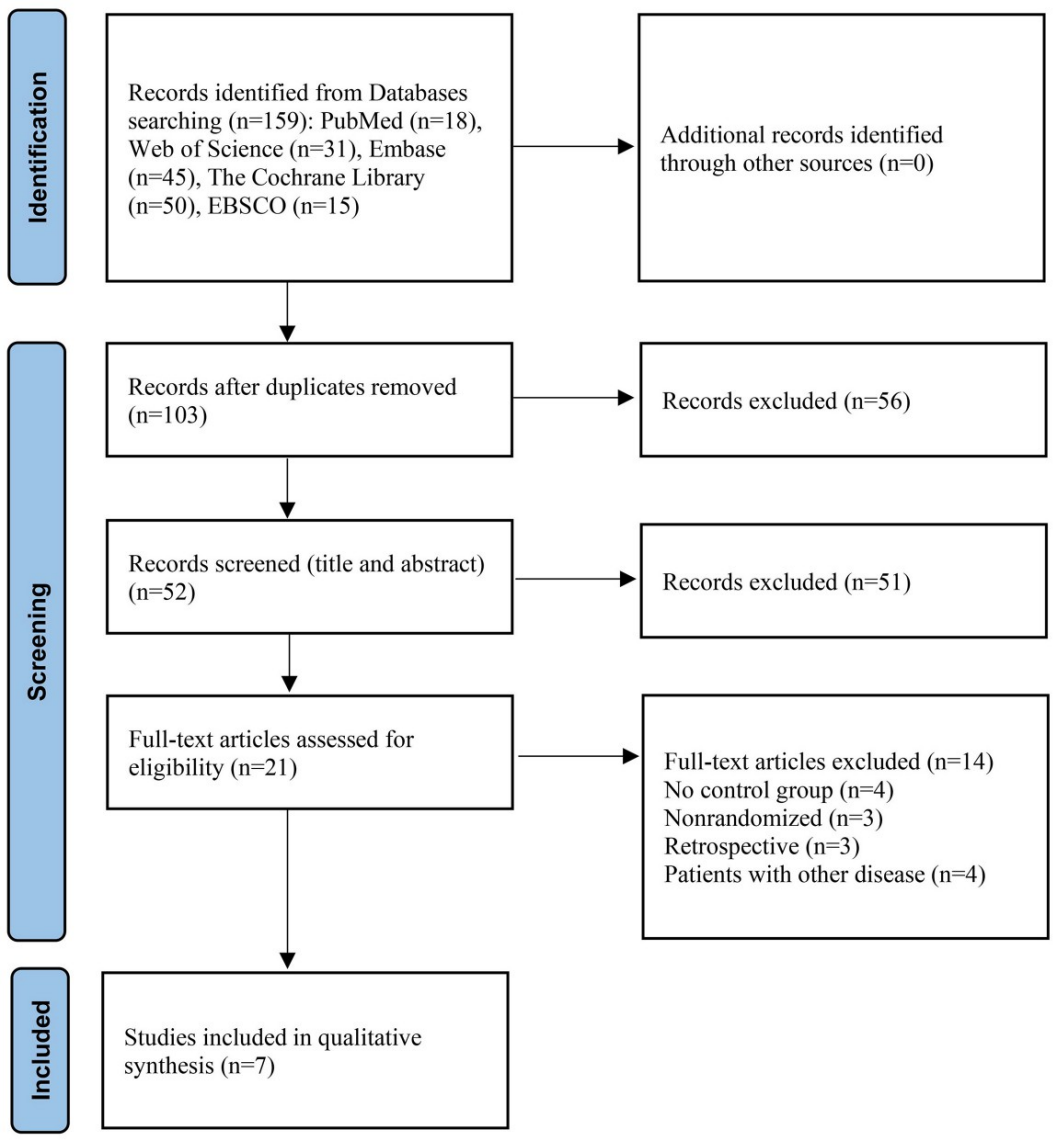
PRISMA (Preferred Reporting Items for Systematic Reviews and Meta-Analyses).

The flow chart (Fig 1) shows 159 potential papers were identified after a systematic search. After removing duplicates and irrelevant records, 21 studies were eligible for full-text reading. Finally, seven RCTs were included in the systematic review [23–29].

### 3.2 Study characteristics

Seven RCTs with 278 patients who underwent ACLR were included: 139 were in the KT group, and 139 were in the control group. Table 1 reports the study participant characteristics. Table 2 presents the study intervention characteristics, including treatment methods and related parameters of the KT group and the control group. Of these studies, 3 reported quadricep strength [23, 27, 29], 2 reported hamstring strength [23, 27], 4 reported knee swelling [24–26, 28], 5 reported knee flexion angle [23–26, 28], 3 reported Lysholm knee function scores [23, 26, 28], and 4 reported VAS pain scores [24–26, 28].

### 3.3 Study quality assessment

**Fig 2:**
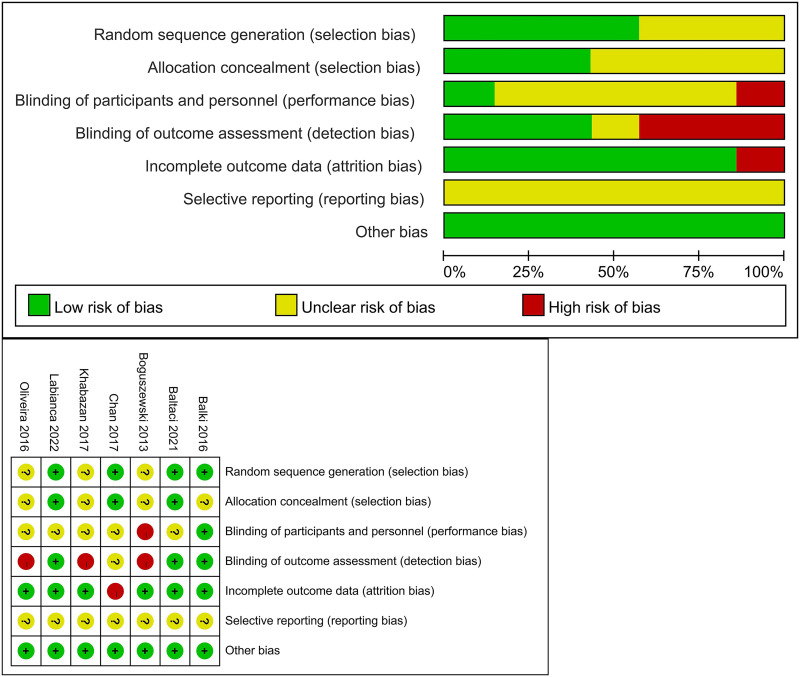
Results of the bias assessment of the included studies.

Four studies described the specific methods used for random sequence generation [23, 24, 26, 28]. Three studies explicitly stated allocation concealment [24, 26, 28]. One double-blind study [23] and two single-blind studies [24, 28] were conducted. One study reported dropout but did not provide a clear reason for the dropout [26]. The existence of other possible biases was unclear (Fig 2).

### 3.4 Clinical results

#### 3.4.1 Quadricep strength

One of three (33%) studies found a substantial increase in quadricep strength associated with the use of KT compared with the control. Khabazan 2017 et al. [27] found a considerable increase in quadricep strength after 24 h of KT application. Conversely, Oliveira 2016 et al. [29] reported testing in the taping state did not remarkably improve quadricep strength in patients after ACLR 12–17 weeks postoperatively. Similarly, Balki 2016 et al. [23] did not find an improvement in quadricep strength with KT.

#### 3.4.2 Hamstring strength

Two of two (100%) studies noted substantial increases in hamstring strength associated with KT. Balki 2016 et al. [23] demonstrated a considerable improvement in hamstring strength after 10 days of KT application compared with the sham KT group. Khabazan 2017 et al. [27] reported a substantial increase in hamstring strength after 24 h KT application.

#### 3.4.3 Knee swelling

Two of four (50%) studies reported KT reduced knee swelling. Boguszewski 2013 et al. [25] showed the application of KT for four weeks in addition to conventional rehabilitation notably relieved knee swelling in patients after ACLR, consistent with the findings of Labianca 2022 et al. [28] However, in the study of Chan 2017 et al. [26], the control group took conventional rehabilitation, and the KT group applied two weeks of KT based on the control group; no group differences in knee swelling were found. In addition, Baltaci 2021 et al. [24] demonstrated no substantial differences between the groups in knee swelling.

#### 3.4.4 Knee flexion angle

Two of five (40%) studies reported substantial improvements in knee flexion angle in the groups that used KT. Balki 2016 et al. [23] showed knee flexion angle was considerably improved in patients who underwent ACLR after 10 days of KT applications compared with the sham KT group. Boguszewski 2013 et al. [25] showed the application of KT for four weeks of conventional rehabilitation substantially improved knee flexion angle in patients after ACLR. However, Baltaci 2021 et al. [24] showed the application of KT for three days did not improve the knee flexion angle compared with the control group. Chan 2017 et al. [26] reported applying KT for two weeks did not considerably improve knee flexion angle in patients after ACLR compared with the control group. Labianca 2022 et al. [28] randomized 52 patients who underwent ACLR into two groups. The control group received conventional rehabilitation, and the KT group underwent KT-based conventional rehabilitation. After four weeks of treatment, no group differences in knee flexion angle were found.

#### 3.4.5 Lysholm knee function score

Three of three (100%) studies found KT did not improve Lysholm knee function scores. Balki 2016 et al. [23] randomly divided 30 patients who underwent ACLR into two groups; the control group received sham KT, and the KT group received KT for 10 days. Both groups then underwent conventional rehabilitation for three months, and the result showed no group differences in Lysholm knee function score. In the study of Chan 2017 et al. [26], the control group underwent conventional rehabilitation, and the KT group underwent KT. No between-group differences in Lysholm knee function scores were found. Labianca 2022 et al. [28] randomly divided 52 patients who underwent ACLR into two groups. The control group received conventional rehabilitation, and the KT group underwent KT-based conventional rehabilitation. No between-group differences in Lysholm knee function scores were found after four weeks of treatment.

#### 3.4.6 VAS pain score

Three of four (75%) studies noted a substantial reduction in VAS pain scores associated with KT. Balki 2016 et al. [23] randomized 30 patients who underwent ACLR into the sham KT group and the KT group. The results showed the KT group had remarkably lower night pain scores on day 10 after treatment compared with the sham KT group. Labianca 2022 et al. [28] randomly divided 52 patients who underwent ACLR into two groups. The control group received conventional rehabilitation, and the KT group applied KT based on conventional rehabilitation. After two weeks of treatment, the VAS pain score of the KT group was substantially lower than that of the control group, and no group difference in VAS pain score was found after four weeks. Boguszewski 2013 et al. [25] showed the application of KT for four weeks in addition to conventional rehabilitation considerably improved the VAS pain score. However, Baltaci 2021 et al. [24] demonstrated no substantial differences between the groups in VAS pain score.

## 4 Discussion

The present study is the first systematic review to investigate the rehabilitative efficacy of KT in patients who underwent ACLR, and seven studies involving 278 participants were included. The results indicate KT may help improve hamstring strength and reduce knee swelling and pain in patients after ACLR. Further studies are needed to determine the effects of KT on quadricep strength and knee flexion angle.

### 4.1 Effect of KT on muscle strength

The results among the included studies regarding the effects of KT on quadricep strength were conflicting. The results of the articles differ from those of studies conducted in populations with chronic musculoskeletal disorders that reported improvements in quadricep strength with KT. Aghapour et al. [30] showed KT substantially improved peak quadricep torque in patients with patellofemoral pain syndrome. The authors attributed this to the reduction in patient pain with KT. In the early postoperative period after ACLR, when patients are in acute pain, KT may improve patient muscle strength by reducing pain. Considering patients are unlikely to experience pain in the mid to late postoperative period, KT would unlikely improve muscle strength by reducing pain. Balki et al. [23] showed the early postoperative application of KT improved hamstring strength; however, it did not enhance quadricep strength, possibly due to arthrogenic muscle inhibition. Arthrogenic muscle inhibition can lead to long-term quadricep strength deficits and poor treatment outcomes [31, 32].

### 4.2 Effect of KT on knee swelling

The management of postoperative swelling is very important in the postoperative rehabilitation after ACLR. This outcome is because swelling causes a decrease in quadricep strength through arthrogenic muscle inhibition, which further causes changes in movement patterns [33]. The tension provided by KT can increase blood circulation and lymphatic drainage, and reduce swelling [34]. However, the efficacy of KT on knee swelling in patients after ACLR remains controversial. Boguszewski 2013 et al. [25] showed a substantial reduction in knee swelling in patients after ACLR with four weeks of taping. Chan 2017 et al.’s [26] study did not show any additional gain of KT in reducing knee swelling, probably due to the short duration of taping. Moreover, the efficacy of KT is influenced by the application protocol, and lymphatic KT may help reduce knee swelling. Białoszewski et al. [35] and Donec et al. [17] reported lymphatic KT can effectively control swelling. Previous studies have shown lymphatic KT can reduce swelling by stimulating the drainage of edema present in the interstitial space to less congested lymphatic channels [33, 36].

### 4.3 Effect of KT on knee flexion angle

The elasticity of the KT enables the joint to achieve full range of motion and does not limit its range of motion [37]. In addition, previous studies have shown KT can improve blood circulation within the taped area and to some extent, enhance the joint range of motion [38]. Of the five studies included, two showed substantial improvement in knee flexion angle with KT. Balki 2016 et al. [23] applied a Y-shaped KT to the quadriceps and hamstring with 25%–50% tension and fan-tapes to the nearby lymph nodes. Knee flexion angle considerably improved in patients who underwent ACLR after 10 days of KT application compared with the sham KT group. Boguszewski 2013 et al. [25] applied a four-week Y-shaped KT to the quadriceps with a tension of 15%–50% and a I-shaped KT between the tibial tuberosity and the femoral condyles with 75%–100% tension. KT considerably improved knee flexion angle in patients after ACLR. However, Labianca 2022 et al. [28] applied quadricep taping with 40% tension to patients after ACLR but did not find any improvement in knee flexion angle after four weeks of treatment. Chan 2017 et al. [26] applied a knee grid-like patch with 10% tension postoperatively to patients after ACLR and also did not find any improvement in knee flexion angle. This outcome implies the efficacy of KT on knee flexion angle may be influenced by the mode of application, and within a certain range, the application of higher tension KT at multiple sites may be more helpful in improving knee flexion angle.

### 4.4 Effect of KT on VAS pain score

Evidence for the effect of KT on musculoskeletal pain is inconsistent. A systematic evaluation of the efficacy of KT in patients with knee osteoarthritis showed KT helps reduce pain [15]. However, this finding is inconsistent with that of Montalvo et al. [39], who included 13 studies that systematically evaluated the efficacy of KT in musculoskeletal disorders. The result showed KT has limited potential for reducing pain. The differences in study results may be due to dissimilarities in disease type and KT protocol. Our findings add to the existing knowledge that KT can positively affect pain reduction in acute musculoskeletal pain (post ACLR). KT can reduce pain by increasing lymphatic drainage and reducing interstitial pressure. In addition, the application of KT instills a sense of confidence and support in patients, psychologically preparing them to return to exercise and reducing their fear of injury. Some scholars believe reducing fear associated with exercise can lessen pain because fear of exercise is the most substantial predictor of pain [40]. Another theory suggests KT reduces pain by promoting pain-inhibitory pathways descending from higher brain centers [39]. KT stimulates the release of analgesic neurotransmitters through a placebo effect [39]. Some authors believe psychological factors largely determine the short-term effects of KT [41].

### 4.5 Clinical summary

To date, limited evidence from several studies has demonstrated KT can improve muscle strength in patients after ACLR, which is largely achieved by reducing knee pain and swelling. A recent systematic review may help confirm this hypothesis. Csapo et al. [42] included 19 studies that systematically evaluated the effect of KT on muscle strength in healthy individuals. The result showed no additional gain in muscle strength with KT. The use of KT as an adjunct in the early postoperative period may contribute to the reduction of pain and swelling, which in turn, may enhance functional muscle performance during testing. Previous studies have also suggested a possible therapeutic time window in which the KT is most effective when applied immediately after surgery [26]. Liu et al. [43] showed the importance of management in pain, swelling, and range of motion in the early postoperative period, during which all parameters influence the next phase of rehabilitation. This outcome may provide some insight that the application of KT in the early postoperative period can reduce pain and swelling, improve joint range of motion, and positively influence later functional recovery. This effect may be influenced by the KT protocol and the intervention time. In addition, the psychological effect of KT is not excluded, and further studies should be conducted to verify these hypothesized mechanisms.

### 4.6 Limitations and strengths

This study has some shortcomings. First, the small number of studies involved in some of the outcome indicators may reduce the reliability of the evidence. Second, the intervention time after ACLR varies in the included literature, which may increase the heterogeneity of the results. The results also support that KT may be more effective for patients in the early postoperative period after ACLR. In addition, the KT protocol was not fully considered, and the treatment effect may vary among taping methods. Considering these reasons, only a systematic review, not a meta-analysis, was performed. This study provides a reference for the clinical application of KT in patients after ACLR. Future studies should establish standardized application techniques and determine the optimal time of application.

## 5 Conclusion

The results of this systematic review indicate KT may help improve hamstring strength and reduce knee swelling and pain in patients after ACLR. Further studies are needed to determine the effects of KT on quadricep strength and knee flexion angle. Considering KT is a low-cost, low-risk treatment, it may be used as an adjunct to facilitate early functional recovery after surgery.

## Supporting information

S1 Checklist PRISMA 2020 for abstracts checklist. (DOCX)

S2 Checklist PRISMA 2020 checklist. (DOCX)

S1 Data Raw data. (XLSX)
